# Choice and Trade-offs: Parent Decision Making for Neurotechnologies for Pediatric Drug-Resistant Epilepsy

**DOI:** 10.1177/08830738211015010

**Published:** 2021-06-02

**Authors:** Viorica Hrincu, Patrick J. McDonald, Mary B. Connolly, Mark J. Harrison, George M. Ibrahim, Robert P. Naftel, Winston Chiong, Armaghan Alam, Urs Ribary, Judy Illes

**Affiliations:** 1 8166 University of British Columbia, Division of Neurology, Department of Medicine, Vancouver, British Columbia, Canada; 2 Faculty of Medicine, Division of Neurosurgery, Department of Surgery, 8166 University of British Columbia, Vancouver, British Columbia, Canada; 3 Department of Pediatrics, Division of Neurology, BC Children’s Hospital, Vancouver, British Columbia, Canada; 4 Faculty of Pharmaceutical Sciences, 8166 University of British Columbia, Vancouver, British Columbia, Canada; 5 Centre for Health Evaluation and Outcome Sciences, St. Paul’s Hospital, Vancouver, British Columbia, Canada; 6 Division of Neurosurgery, 483367 Hospital for Sick Children and Toronto Western Hospital, Toronto, Ontario, Canada; 7 Department of Neurosurgery, 12328 Vanderbilt University Medical Center, Nashville, Tennessee, USA; 8 Weill Institute for Neurosciences, Department of Neurology, Memory and Aging Center, 550067 University of California San Francisco, San Francisco, CA, USA; 9 Behavioral & Cognitive Neuroscience Institute, 1763 Simon Fraser University, Burnaby, British Columbia, Canada

**Keywords:** epilepsy, child, neurotechnology, neuroethics, neurosurgery, decision making, parents

## Abstract

This qualitative study investigated factors that guide caregiver decision making and ethical trade-offs for advanced neurotechnologies used to treat children with drug-resistant epilepsy. Caregivers with affected children were recruited to semi-structured focus groups or interviews at one of 4 major epilepsy centers in Eastern and Western Canada and the USA (n = 22). Discussions were transcribed and qualitative analytic methods applied to examine values and priorities (eg, risks, benefits, adherence, invasiveness, reversibility) of caregivers pertaining to novel technologies to treat drug-resistant epilepsy. Discussions revealed 3 major thematic branches for decision making: (1) features of the intervention—risks and benefits, with an emphasis on an aversion to perceived invasiveness; (2) decision drivers—trust in the clinical team, treatment costs; and (3) quality of available information about neurotechnological options. Overall, caregivers’ definition of treatment success is more expansive than seizure freedom. The full involvement of their values and priorities must be considered in the decision-making process.

Technological advances over the past several decades have resulted in novel interventions available to pediatric neurosurgeons to treat drug-resistant epilepsy. These include deep brain stimulation, vagus nerve stimulation, responsive neurostimulation, MRI-guided laser interstitial thermal therapy, and stereotactic radiosurgery.^1^ Only limited knowledge is available about the views of caregivers and parents about such interventions,^2^ and their decisions are often made in the context of incomplete evidence regarding the efficacy, safety, and long-term side effects of treatment.^3^ Conventional resective neurosurgery is an effective procedure for about one-third of all children with drug-resistant epilepsy,^4^ but its invasive and irreversible nature can make it a daunting choice for caregivers who bear the burden of decision making. Contextual factors^5^ and the imperative for timely intervention further^6,7^ influence the ethical magnitude of benefit weighed against associated risks perceived by parents of children with drug-resistant epilepsy.

This research completes a suite of studies with key stakeholders^8,9^ that share the common goal of addressing shared decision making and the vulnerability of children with drug-resistant epilepsy whose bodies, brains, and experiences are still evolving.^8-10^

## Materials and Methods

### Design

Using purposive sampling methods, caregivers of children with drug-resistant epilepsy who had undergone a surgical intervention for drug-resistant epilepsy were recruited through clinics with an established epilepsy surgery program in the eastern and western regions of Canada and the United States. Centers were chosen for their high volume of epilepsy surgeries and early adoption of novel surgical interventions for drug-resistant epilepsy.

We conducted 3 focus groups at 3 separate sites: SickKids Hospital Toronto, Ontario, BC Children’s Hospital, Vancouver, BC, and Monroe Carell Jr.’s Children’s Hospital, Vanderbilt, Nashville, Tennessee. Owing to COVID-19, we conducted 2 individual interviews instead of the fourth in-person focus group planned for UCSF Benioff Children’s Hospital in San Francisco, California. Focus groups had a preset date and time and were advertised via posters, pamphlets, and advocacy websites. Physicians were invited to inform families about the study and offered contact information to the local Research Coordinator for follow-up and consent if interested. One parent per family participated.

### Setting

Focus groups were led by the principal or co–principal investigator, a local collaborator at the remote sites, and a researcher responsible for taking field notes. Family focus groups were held in hospital conference rooms, beginning with refreshments, a review of consent, answers to questions from participants, and a 5-6-minute informational video about neurotechnology for drug-resistant epilepsy. Individual interviews followed similar consent procedures and were led by the principal investigator and one local collaborator over Zoom. All sessions were audio recorded.

### Materials

We collected key demographic indicators of age, gender, educational level, ethnicity, experience with drug-resistant epilepsy, and medications for each participant. The video provided examples of currently used surgical interventions, presented neuroethical issues, such as risk, benefit, and reversibility, and discussed compliance requirements associated with treatment options.

### Data Analysis

Following the protocol for data analysis reported in McDonald et al,^8^ focus group and interview audios were transcribed, made software-ready for NVivo (QSR 12), and analyzed using qualitative content analysis.^11-13^ Results were interpreted using a pragmatic neuroethics framework,^14^ respecting the plurality of views and focusing on evidence to support practical recommendations. Two researchers (V.H., A.A.) independently read the transcripts and coded them line-by-line to identify major themes. V.H. and A.A. co-coded 15% of the transcripts to test for interrater reliability. Discrepancies were discussed until consensus was reached. A Cohen kappa of 80% indicated a high intercoder reliability. A priori categories were used to construct the codebook, and additional themes were later incorporated as they emerged through inductive and deductive analysis of the transcripts. Illustrative quotes are used here to elaborate on salient thematic points, and ellipses applied for clarity and readability.

We visualized the results quantitively into a pedigree structure based on data from the focus groups: major thematic branches (topmost level), major themes, and minor themes. Major themes constituted the top 50% most frequently coded topics in each thematic branch. Because there were only 2 interviews, we sought qualitative overlap where applicable to major themes. The label of minor represents relative quantitative status, not importance. Minor themes identified as such add qualitative depth or insight.

## Results

### Participants

Of the 22 participants, 82% identified as women. Median age was 46 years. All but 3 participants were married. Eighteen participants had at least some college or university education. Seventeen participants were white; 2 were from Asian, 1 Latin, and 1 American Indian or Alaska Native and White (mixed race) backgrounds. Sixty-three percent of participants had a household income greater than $75 000, which is high compared with the national medians for each country.^15,16^ Table 1 summarizes the reported demographics of their drug-resistant epilepsy–affected children.

**Table 1.** Demographics of the Children of Participants.

*Age of child (y)*	
Median	13
Range	2-33
*Gender of child*	
Female	10
Male	12
*Age of child at diagnosis (y)*	
Median	3
Range	Newborn to 19
*Time since last intervention (months)^a^*	
Median	9.5
Range	0 to 108
*Treatment history^b^*	
Neurotechnology	9
VNS	7
RNS	1
LITT	1
Open surgery (includes callosotomy, craniotomy, hemispherectomy, cortical resection/lobectomy/lesionectomy)	10
Medication	16
Ketogenic diet	3
Unreported	1

Abbreviations: LITT, laser interstitial thermal therapy; RNS, responsive neurostimulation; VNS, vagus nerve stimulation

^a^ Four participants did not report time since last intervention.

^b^ Most participants reported multiple treatment types in treatment history, but not all participants identified the specific surgical intervention(s) their child underwent.

### Themes

**Figure 1.**
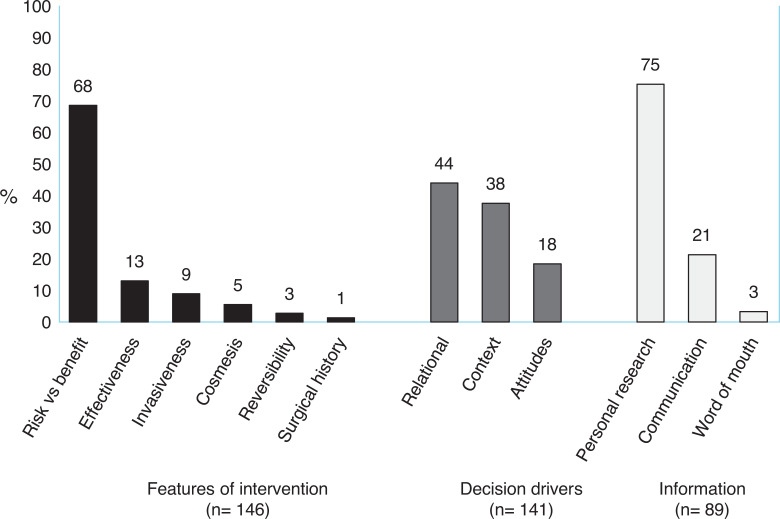
Major themes under each thematic branch.

**Figure 2.**
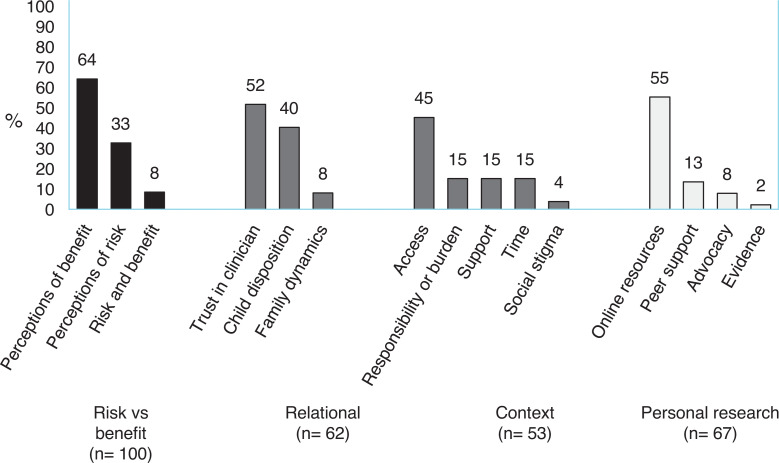
Subthemes comprising major themes.

Focus group and interview analysis revealed 3 major thematic branches: (1) features of the intervention, (2) decision drivers, and (3) sources of information. See Figures 1 and 2.

### Features of the Intervention

When weighing novel neurotechnological treatments, caregivers identified intervention-specific features that impact their decision making. Risk versus benefit was a major theme across all focus groups and interviews. Parents more frequently discussed the perceived benefits of the treatment over the risks, with seizure profile being the expected impetus for seeking treatment, but consistently emphasized that benefits and risks should be weighed against one another.

However, parents emphasized the importance of improving the quality of life of their child overall as treatment exploration progresses—not just seizure freedom—which overlaps with other related factors, such as independence, cognitive function and behavior, and freedom from medication. One parent elaborated on the observed difference between treatment priorities compared to the physicians:

[One thing] surprised me…the doctors were always just concerned with treating the seizures.…The rest of his waking hours we got a whole ton of other problems.…“Look, the little man has his side effects. He goes into rages, he’s punching walls.”…[The doctors] they’re like, “No, don’t worry about that…Let’s just treat the seizures.” (Focus Group 2 Participant, Canada)

Another perceived benefit mentioned across all focus groups was the renewal of hope with the prospect of a novel intervention. Parents expressed a general open-mindedness and willingness to try a novel neurotechnology if there is the potential for any improvement to seizures and overall health and well-being.

On perceived risks, parents described a familiarity with risk in all possible avenues of treatment. However, parents recognized that this familiarity is sometimes harmful, dulling the perniciousness of ongoing seizures. Some parents reported only realizing the dangers of doing nothing when their child became injured following a seizure, or after speaking with their physician. One parent said the following:

I didn’t understand what the risk was, the seizures themselves…it was getting worse and we were getting more used to it.…The more normal it was, the less we cared that it was happening until someone really hardline told me what the outcome was of doing nothing. And I don’t think that was obvious to me at all. I was more prone to research the risks of intervention than I was non-intervention. (Focus Group 2 Participant, Canada)

Parents across all focus groups described a strong aversion to invasive procedures, and conventional epilepsy surgery in particular. For some parents for whose children conventional surgery was an option, the treatment journey began with a less invasive neurotechnology (eg, vagus nerve stimulation) that transitioned to more invasive procedures (eg, temporal lobectomy) as needed.

### Decision Drivers

Decision drivers are the conceptual or practical tools that parents used to decide on whether to choose a neurotechnology. There were 2 major themes under this thematic branch; the first one—relational—encompasses the interpersonal relationships with the physician and clinical team, between parents and their child, and within family dynamics. Parents frequently and emphatically identified trust in the clinical team as paramount to their final decision. Fundamental aspects of trust include the trajectory of relationships over time and reassurance about the procedure. In deference to the team, one participant stated:

We have relied so heavily on the expertise of the doctors that we spoke to and we allowed them to convince us—not that we were totally naïve—but they were the experts. (Focus Group 3 Participant USA)

The disposition of the child was a relational subtheme discussed primarily in the Canadian focus groups. Disposition refers to the capacity, preferences, and values of the child in treatment decisions.

Context was the second major theme under decision drivers, and includes environmental (ie, access) or external factors or pressures that influence caregiver decision making for epilepsy treatment. Ability to pay was a key subtheme for treatment access. Access to treatment, whether due to availability at a specific center or ability to pay, fundamentally impacts decision making. Discussions of access in the US focus groups and interviews focused on the complexities of a multi-payer system:

Two days before we were supposed to have the procedure,…we were packed to go, and my insurance denied the RNS [responsive neurostimulation] and they cancelled the procedure. (Interview 001, USA)

Some caregivers reported no financial barriers to treatment. Other parents expressed fears over the possibility of future cuts to health insurance and discussed moving out of state, even out of country (eg, to Canada) to ensure continued access to health care. Distance was not identified by parents as a barrier. Overall, parents were willing to do or pay anything to help their child:

Even if cost was a factor, for us it wouldn’t be a factor, we just do what we got to do to take care of the things that our kids need. (Interview 002, USA)

Under context, subthemes of responsibility and burden, time, and support were raised. Caregivers across all focus groups reported the heavy emotional burden of treatment decisions, often weighing the immediate needs of their child today versus the future. Some parents noted that multidisciplinary support, especially psychological support, would help to alleviate these additional stressors. Religious faith was a minor but salient subtheme discussed exclusively by participants in the US focus group.

### Information

How parents gathered and evaluated information during the decision-making process was the third major thematic branch. Personal research was conducted primarily through online sources, such as social media, videos, documentaries, academic articles, device manufacturer sites, and usually with the use of popular search engines. Although parents identified academic articles as likely more credible sources of information, they noted that jargon is difficult to understand. Social media is a useful way to connect with other families affected by epilepsy, for both information-gathering and community support. Parents identified a need for objective informational resources directly from hospitals or epilepsy centers, such as a centralized webpage, a collection of frequently asked questions, and printouts or informational pamphlets to take home. For emerging neurotechnologies, questions were specific, such as knowing when to change the battery. One participant noted the pitfalls of information overload on the Internet:

[Once the information is] on the Internet, it gets lost.…we have to decipher whether it’s real. [Getting] information from you [the hospital] directly…it would make me way more comfortable. (Focus Group 1 Participant, Canada)

Parents also reported feeling overwhelmed by the influx of new terminology in meetings with the clinical team, processing not only the facts but the reality of the diagnosis:

It was just all this information that they’re throwing at you…it was overwhelming. (Focus Group 2 Participant, Canada)

Overall, parents reported that their physicians were thorough and reassuring when discussing treatment options with them. Having methods of communication that are inclusive to different communication styles is positive:

The main thing for me as a parent was…not feeling intimidated to ask as many questions as I needed to and being offered over and over again the opportunity to ask questions. (Interview 001, USA)

## Discussion

This qualitative study provides insight into the decision making of caregivers of children with drug-resistant epilepsy when considering a neurotechnological option for treatment. Results suggest that the process of finding an effective treatment is not linear. Caregivers’ state of readiness to make treatment decisions is affected by features of the intervention—primarily benefits and risks—relational and contextual decision drivers, and information acquired throughout the treatment journey. As others have reported, when weighing the benefits and risks, parents focus on benefits of novel neurotechnologies both inside and outside of seizure control –specifically factors pertaining to quality of life, such as mood and independence.^17-20^

The embeddedness of risk in decision making is an ethical consideration that parents expressed for all possible treatment options. Risk was a constant companion to benefit, whether from the seizures or the treatment. In contrast, the perceived benefits of a novel neurotechnology open hope for autonomy (eg, driving) and social interactions (eg, blending in).

Caregivers’ practical acceptance of risk contrasts their immoveable dislike of invasiveness. Risk was separated from invasiveness in the results, because these concepts are not necessarily synonymous. However, parents described invasiveness in the context of treatment as an almost superordinate risk, preferring minimally invasive neurotechnological interventions as a first choice. Related findings on invasiveness or fear of surgery are also documented elsewhere in epilepsy literature.^17,21^ By contrast, some parents unknowingly minimized the risks of the seizures themselves, including sudden death, describing a paradoxical acclimation to the seizures over time. This uneven prescription of risk has ethical implications for the perceived benefit-risk ratio of caregivers and the grounds for desirability of novel neurotechnological treatments.

Caregivers identified several relational and contextual factors that were important for treatment decisions. On a relational level, they value their child’s preferences for treatment. They place great trust in the expertise of the clinical team, and trust is especially strong when a reassuring relationship is built over time. Access to neurotechnology is a potential barrier due to insurance coverage issues in the United States. In contrast, our previous work identified that access to neurotechnology in the single-payer public Canadian setting is more dependent on programmatic government funding.^8^ However, neither cost nor distance were considered deal-breakers for parents’ willingness to do anything that might help their child. Indeed, an emphasis on a holistic approach to “anything that would help” has also been reported for parent perspectives of emerging neurotechnologies for their children with ADHD.^22^ Finally, parents mentioned a need for greater psychological and emotional support during the decision-making process, which they rightly note as being difficult and highly stressful.

Following diagnosis, caregivers reported going through an information-seeking phase.^23^ Initial visits with the clinical team were overwhelming and information-dense, sparking the need to do personal research. Consistent with other studies of personal research by parents of children with chronic illness or disability, participants used online sources for information.^24,25^ They expressed a need for a single legitimate source of information about drug-resistant epilepsy and neurotechnology that is accessible, authoritative, and comprehensive. Parents were not confident in their ability to identify reliable sources of information on the Internet and desire objective and reliable sources of information directly from the institution where their child will receive treatment. In addition, interactions with the clinical team were described as most helpful when communication channels are open and concerns thoroughly addressed without bias.^20^

### Comparison with Other Studies

Although earlier studies investigated the perspectives of caregivers on conventional resective epilepsy surgery,^17,21,23,26-28^ this is the first qualitative, multisite, neuroethical inquiry of caregivers’ decision making on novel neurotechnologies as treatment. The views of physicians were recently captured in a series of publications assessing novel neurotechnologies for children with drug-resistant epilepsy^8^ that differ from caregivers in some key ways. First, physicians’ primary goal is to achieve seizure freedom, whereas caregivers have a more expansive definition of treatment success that includes the various factors improving the quality of life of their child. Second, information-gathering means something different to parents and clinicians. For physicians, information is formalized in the language of evidence. As with other neurologic and neurodevelopmental disorders, caregivers seek and learn about drug-resistant epilepsy and treatment from both expert and nonexpert points of view,^29^ and must sift through information of varying levels of quality and authenticity.^24,30^ Third, physicians are aware of parents’ dislike of invasiveness, but are not necessarily against invasive procedures as long as existing evidence supports good outcomes. The voice of the child in the decision-making process is important to everyone.

### Implications of this Study

There are 4 potential areas of ethical vulnerability that require special care for caregivers faced with choices about a novel neurotechnology for their child with drug-resistant epilepsy (Table 2):

**Table 2.** Ethics Concerns Related to Areas of Vulnerability for Caregivers in the Decision-Making Process.

Area of vulnerability	Ethics concerns
Stage of readiness/Difficulty of decision	-Early in information-gathering phase -Unaware of risks of ineffectively treated epilepsy (ie, paradoxical acclimation to seizures) -No established epilepsy support networks -Emotional or psychological needs not met
Aversion to invasiveness	-Decision based on fear or preconceived notions of treatment type
Access to treatment	-Treatment delays and risks of untreated epilepsy -Implications for justice; certain populations unable to access neurotechnology
Access to reliable information	-Information from sources with a conflict of interest -Information from unreliable sources -Implications for informed consent

- *Stage of readiness/Difficulty of decision*—Physicians must attend to the range of factors that affect the readiness of caregivers at different points in the decision-making process.
- *Aversion to invasiveness*—Perceptions of invasiveness and risk by caregivers depart from those of clinical care providers and may skew accurate evaluation of the risk-benefit ratio of different neurotechnologies.
- *Access to treatment*—Barriers to access have a direct impact on treatment delays and have implications for justice at a societal level.
- *Access to reliable information*—Reliable information resources are needed to avoid language, external pressures, or conflicts of interest^31^ that may steer caregivers toward potentially inappropriate treatment.

### Limitations

Although we reached thematic saturation in the analysis of the data, the English-speaking sample size is small and has limited ethnic, racial, socioeconomic, and cultural diversity. Transferability of the knowledge to other populations and those with different cultural knowledge is methodologically appropriate, but generalizability is not. Timing of intervention discussions with parents is noted as critically important in the pediatric epilepsy literature^21,28^; however, age of the child at time of intervention was not captured in this study. Because the focus of this study was on neurotechnological interventions, we cannot report the precise eligibility or timeline surrounding the children’s medication regime. The views of families who could not access neurotechnology, declined neurotechnology, chose an alternative, or did not access any drug-resistant epilepsy treatment was out of scope for this study. Future studies are needed to investigate the role of socioeconomic and other demographics factors on choice and the interplay with caregiver and clinician outcome measures.

## Conclusions

The burden of decision making for neurotechnology to treat drug-resistant epilepsy is defined by the continuity of readiness and receptivity of caregivers to options that are guided by the medical condition of affected children, the context in which they live, their preferences, and the desire for autonomy. The benefit-risk ratio dominates technical aspects of the decision-making process. Trust in the clinical team and the availability of trustworthy information are vital to the success of these decisions.
